# Supplementary material for: Causal Relationship between Aging and Anorexia Nervosa: A White-Matter-Microstructure-Mediated Mendelian Randomization Analysis
Source: Biomedicines. 2024 Aug 16;12(8):1874. doi: 10.3390/biomedicines12081874 (PMC11351342; doi:10.3390/biomedicines12081874)
Supplement: Supplementary file 1 [file biomedicines-12-01874-s001.zip › Supplementary Figure/Figure S4/Singe SNP plot of MR analysis for TL to ubm-b-1889.pdf]

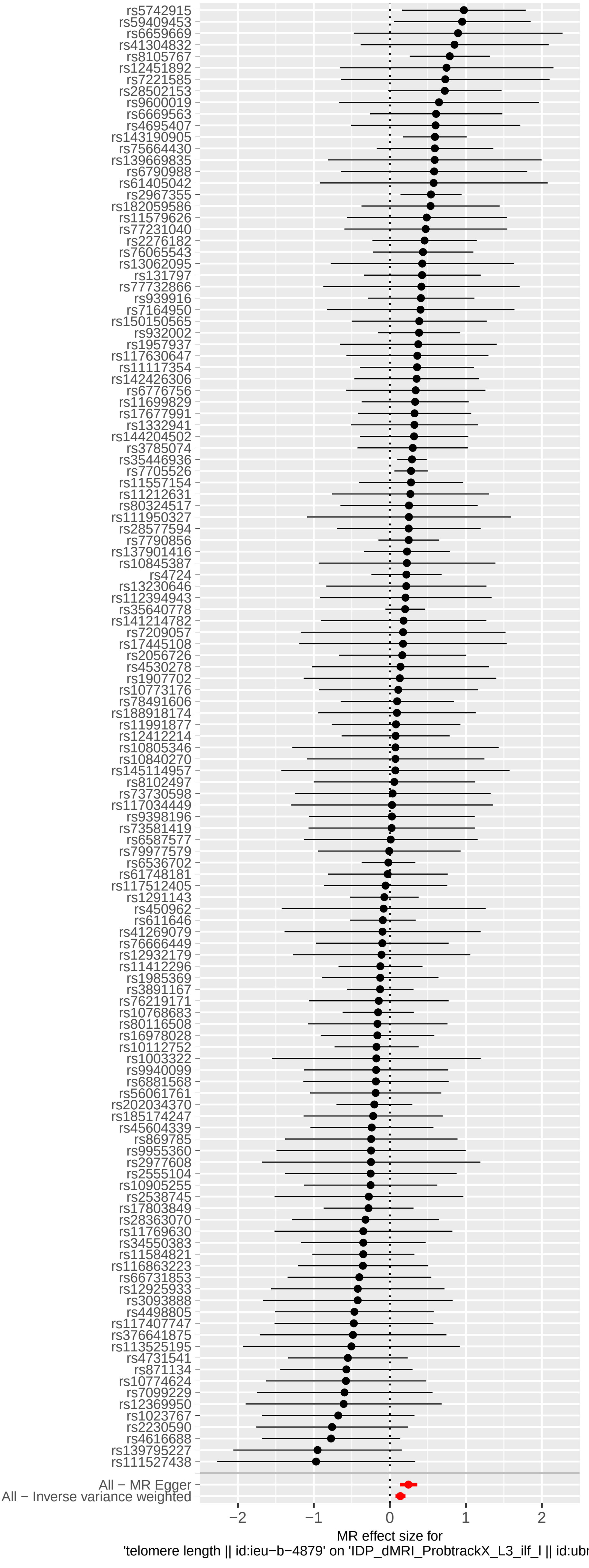

rs5742915
rs59409453
rs6659669
rs41304832
rs8105767
rs12451892
rs7221585
rs28502153
rs9600019
rs6669563
rs4695407
rs143190905
rs75664430
rs139669835
rs6790988
rs61405042
rs2967355
rs182059586
rs11579626
rs77231040
rs2276182
rs76065543
rs13062095
rs131797
rs77732866
rs939916
rs7164950
rs150150565
rs932002
rs1957937
rs117630647
rs11117354
rs142426306
rs6776756
rs11699829
rs17677991
rs1332941
rs144204502
rs3785074
rs35446936
rs7705526
rs11557154
rs11212631
rs80324517
rs111950327
rs28577594
rs7790856
rs137901416
rs10845387
rs4724
rs13230646
rs112394943
rs35640778
rs141214782
rs7209057
rs17445108
rs2056726
rs4530278
rs1907702
rs10773176
rs78491606
rs188918174
rs11991877
rs12412214
rs10805346
rs10840270
rs145114957
rs8102497
rs73730598
rs117034449
rs9398196
rs73581419
rs6587577
rs79977579
rs6536702
rs61748181
rs117512405
rs1291143
rs450962
rs611646
rs41269079
rs76666449
rs12932179
rs11412296
rs1985369
rs3891167
rs76219171
rs10768683
rs80116508
rs16978028
rs10112752
rs1003322
rs9940099
rs6881568
rs56061761
rs202034370
rs185174247
rs45604339
rs869785
rs9955360
rs2977608
rs2555104
rs10905255
rs2538745
rs17803849
rs28363070
rs11769630
rs34550383
rs11584821
rs116863223
rs66731853
rs12925933
rs3093888
rs4498805
rs117407747
rs376641875
rs113525195
rs4731541
rs871134
rs10774624
rs7099229
rs12369950
rs1023767
rs2230590
rs4616688
rs139795227
rs111527438
All − MR Egger
All − Inverse variance weighted
−2
−1
0
1
2
MR effect size for
'telomere length || id:ieu−b−4879' on 'IDP_dMRI_ProbtrackX_L3_ilf_l || id:ubr
